# Epidemic Fever

**Published:** 1820-06-01

**Authors:** 


### EPIDEMIC FEVER.

Hinc hominum pecudumque lues, hinc pestifer aer. CLAUD.

1. *Medical Report of the Fever Hospital and House of Recovery, Cork Street, Dublin, for the Year ending the* 4*th of January* 1819. By RICHARD GRATTAN, M. D. Fellow and Censor of the King and Queen's College of Physicians, &c. &c. Octavo, pp. 123. Sewed, 1819.

2. *Medical Report of the Fever Department in Steevens' Hospital; containing a brief Account of the late Epidemic in Dublin, from September* 1817 *to August* 1819. By JOHN CRAMPTON, M. D. Physician to Steevens' Hospital, &c. &c. Octavo, sewed, pp. 63. Dublin, 1819.

As in the Journey through life, we frequently gather our honey from thorns, so, in science, truth often springs from error. In medicine, this is particularly illustrated in numerous instances. If the arguments of some distinguished physicians, in favour of the *topical* nature of fever, have not brought conviction, they have been very useful in exciting to such a rigid investigation as has fully proved that, though local inflammation be not the *cause*, it is very frequently the *consequence*, or concomitant of fever, especially in its severer forms and ulterior stages.

When we consider, indeed, how very rare it is to find a constitution, in the present æra of the world, so nicely poised, that every organ and function is in a state of integrity, and developed proportionally to other organs and functions, we cannot wonder that, in nine cases out of ten, a general febrile movement in the system soon takes a local direction, determined by some pre-existing lesion of function or structure. Were ten men, in apparent health, exposed naked, on the buring sands of Arabia, to the fervour of a tropical sun, until a general excitement was kindled up, and death ultimately ensued, nine of them, if not the whole, would exhibit marks of local congestion or inflammation, on dissection. Yet, surely, no man would say that these *topical* affections were the *cause* of the general excitement, although they might probably be the principal occasion of the fatal termination.

The doctrine of a *particular* topical inflammation, (as of the brain or digestive apparatus) being always the cause of fever, is still less tenable. Indeed, when a house divides against itself, it must fall; and the exclusive hypotheses of Clutterbuck and Broussais have necessarily proved fatal to each other. Our brethren in Ireland have poured a flood of light upon the nature and seat of fever, and confirmed, by an unbounded scope of observation, certain rational and enlightened views of the disease, which several individuals had previously taken, though in a more limited sphere of experience. In the fourth number of our Quarterly Series, we gave a pretty full analysis of the Irish Fever Reports up to that time, and we are now to lay before our readers an account of the two Pamphlets at the head of this article, whose perishable forms will induce us to give to their valuable contents an extent of record in this Journal, proportioned rather to their merit than to their magnitude. All details of a local or economical nature, however, must be passed over, for obvious reasons; and nothing extracted beneath the level of general interest. Yet, small as these Reports are, we shall not be able to do justice to them, even in an extended article. We shall take them in the order in which they stand.

Dr. Grattan observes, that the domestic history of Ireland affords no instance of an epidemic fever having followed the same steady and long-continued course which it has of late years pursued. Into three Institutions only, 25,336 fever patients were admitted, between the years 1810 and 1814, evincing that this fever had long prevailed, and was gathering strength long before the date of what is called the present epidemic. Between the 1st of Sept. 1817, and the end of May 1819, the admissions into the *Dublin* Hospitals amounted to 39,598; of whom 1,857 died, or about 1 in 22, a very small ratio of mortality. The records of this wide-spreading epidemic incontestibly prove, that the commonly specified etiology, bad harvests, and unwholesome food, is exceedingly defective; and that we are yet ignorant of the real effective causes of epidemic diseases. Dr. Grattan, therefore, well observes, that "we should not affect too much simplicity, nor should we endeavour to ascribe to a single cause, that which has probably been the result of several."

"That fever is contagious, is now so well established, that I believe there are few physicians so much the slaves of theory as to doubt the accuracy of an opinion, the truth of which is generally admitted." 25.

But Dr. Grattan wisely avoids inferring, that because contagion will produce fever, all fevers must be produced by contagion, as some have done. We entirely agree with our intelligent author, that in the course of those changes which take place without interruption, amongst the various kinds of organized matter, products may, and do occasionally arise, which never before existed; and which, when applied to the system, are capable of producing diseases of a character totally different from any hitherto observed. In this way only can we account for new diseases.

"And if it be possible for any cause, or combination of causes, to occasion a new disease, why may not that disease be contagious, and be again called into existence, independently of contagion, by the same causes which first produced it?"

We see no reason, indeed, to the contrary; for, as Dr. O'Brien has well observed, "if the opinion that contagion is the only source of typhus were true, we must be reduced to the necessity of supposing that all contagious diseases were derived from Adam himself."

Our author also is of opinion, that a fever once generated, from whatever cause, may, under certain circumstances, become contagious; in which creed, we believe all the most enlightened and experienced practitioners are agreed. Next to contagion, Dr. Grattan considers the *distressed state* of the general population of Ireland, to be the great exciting cause of fever. Whether this result from the miseries of war or domestic misfortune is, he thinks, immaterial.

"In either case, that peculiar state is induced which both calls contagion into existence, and which, at the same time, renders the human frame more susceptible of its influence. When distress prevails, the depressing passions must lower the energy of the nervous system, and dispose it to the reception of contagion, while the causes likely to generate contagion, concur at the same time to produce it. To the connection, and reciprocal action of both of these, the presented epidemic is to be ascribed." 40.

Atmospherical vicissitudes, intemperance, fatigue, suppressed perspiration, the depressing passions, &c. when excessive, will induce fever; and under these circumstances, the accumulation of animal effluvia, in filthy, crowded, and ill-ventilated dwellings, will generate contagion, which, of course, accelerates the march of the epidemic.

Dr. Grattan observes, that although the writings of late authors have pretty uniformly agreed in the propriety of blood-letting in fever, and have therefore "contributed to the improvement of medicine, by dispelling prejudices, and removing opinions which, though now admitted to be erroneous, were, until lately, very generally acted upon in practice;" yet, he thinks, that in this great revolution, zeal has, in some degree, usurped the place of prudence, and an anxiety to overturn the tottering remnants of a baseless system, has occasioned some injury, by involving in the same ruin, previous acquisitions of real value. Here Dr. G. enters into a critical examination of Dr. Clutterbuck's late work on fever, and condemns, from experience, the practice which Dr. Clutterbuck has recommended, of bleeding in the incipient movements of fever, anterior to evident re-action.

"These and similar positions, says our author, are of a nature so serious, that they call for the most mature and deliberate consideration before we can be expected to afford our assent to them. The doctrine that *all* fevers are essentially inflammatory, has not yet received sufficient confirmation, either from pathological reasoning, or anatomical research, to satisfy us of its accuracy; and until it shall be clearly proved that such is the case, those physicians who entertain a contrary opinion, and refer the immediate cause of fever to a disordered state of the nervous system, altogether distinct from inflammation, have at least an equal right to presume that their theory is correct." 79.

Anatomical investigations having shown that, in numerous instances, the scalpel can detect no appearance of inflammation in the brain, it follows, of course, Dr. G. observes, that the doctrine of topical inflammation as the cause, is quite untenable.

"I do not pretend to deny that the brain, or any other organ of the body, can be occupied by inflammation during the continuance of fever. On the contrary, I am persuaded it often happens, that in the course of the fever, congestions of blood, and its unequal and irregular distribution, do actually occur in different organs, varying in degree from the lowest kind of passive congestion to the most marked and decided form of active inflammation. In every instance, however, this congestion, or active inflammation, of what kind soever it may be, is secondary, and depends on the peculiar state of the nervous system, the only part primarily and essentially affected. In what this condition of the nervous system consists I do not presume to explain, and I fear it is one of those secrets which nature will for ever conceal from us. Anatomical research cannot elucidate it, in as much as it is a modification of that unknown influence, the principle of life itself, which must cease to exist before the anatomist can have commenced his inquiries." 80.

The *post mortem* appearances in the vascular system, Dr. Grattan thinks, are sometimes fallacious. Distention of the cerebral vessels, in the corpse, is not an indubitable proof of congestion during life. The elastic and tonic power of the arteries empties them, as soon as the heart ceases to act, and the least resisting capillaries often receive a share of the arterial blood, giving them the *appearance* of having been previously congested or even inflamed. We have often inculcated the necessity of always coupling the symptoms during life with appearances on dissection, otherwise we shall frequently be led into false conclusions. We are glad to find our author of a similar opinion. We need not say how congenial the following sentiments are with those which we have long maintained in this Journal.

"The doctrine which teaches that fever is a disease of the nervous system, and at the same time admits that this diseased action does often occasion local inflammation, seems to be nearer to the truth than if we were to ascribe fever to either of these causes exclusively. It embraces both theories, and in a practical point of view, comprehends every case, and every variety of treatment which can be employed in the management of fever. From an attentive consideration of the subject, and from such observations as I have been able to found on my own experience, I can affirm, that it leads to the most successful practice in the fever which now prevails in Ireland, and which is identical in its nature and symptoms with the ordinary fever that has always existed in this country. Its obvious tendency is to substitute a rational treatment for blind empiricism, to cause the physician to reflect before he prescribes, and while it inculcates prudence, it by no means precludes him from adopting the most active measures in those cases in which they may appear to be necessary." 82.

Speaking of the large blood-lettings which Dr. Clutterbuck recommends in the incipient movements of fever, our experienced author observes thus:

"In the fevers which I have had an opportunity of observing, the practice recommended by Dr. C. is totally inadmissible; and therefore, even admitting that his theory is much more probable than it appears to be, still the cases to which it is altogether inapplicable are so numerous, that it should be received and acted upon with the greatest caution. I cannot agree with Dr. C. in thinking that early blood-letting, when used with tolerable freedom, may be considered an almost certain *preventive* with regard to *symptoms of malignity.* My experience is quite at variance with this opinion; and I can state, that some of the most malignant cases of fever, which I have ever witnessed in the hospital, had been largely blooded by irregular practitioners previously to their admission. Amongst such patients the mortality is always greater; and I have remarked that, independently of their having a worse chance of recovery, they die within a much shorter period than others who have not been so actively treated." 83.

Dr. Grattan wishes it to be distinctly understood that he is here speaking only of the epidemic fevers of Ireland—"*Hibernos afficientes et in Aere Hiberno.*" He is persuaded that the fevers of different climates and countries differ, in numerous instances, so much from each other, as to require very different modes of practice.

During the last year which this Report embraces, there passed through the Institution to which Dr. Grattan is physician, 7608 patients. The following table exhibits a view of the complicated cases, and the extent of blood-letting employed.

" Table of the number of fever cases, accompanied with complicated symptoms, as they occurred in each month, explaining the part of the system most affected, and affording a general view of the number of patients who were blooded, of the total quantity of blood taken, and of all the deaths."

| Months. | No. of cases with complicated symptoms. | Chest. | Bled. | Total oz. | | Bled. | Total oz. | Abdom. | Bled. | Total oz. | Total Deaths. |
|---|---|---|---|---|---|---|---|---|---|---|---|
| January | | | | | | | | | | | |
| February | 52 | 34 | 17 | 93 | 19 | 8 | 37 | 3 | 3 | 32 | 3 |
| March - | 70 | 41 | 36 | 164 | 27 | 18 | 107 | 3 | 3 | 16 | 5 |
| April - | 58 | 37 | 9 | 61 | 32 | 11 | 66 | 2 | 2 | 12 | 2 |
| May - - | | | | | | | | | | | |
| June - | | | | | | | | | | | |
| July - | 66 | 26 | 11 | 52 | 42 | 5 | 25 | 0 | 0 | 0 | 2 |
| August - | 61 | 32 | 7 | 42 | 37 | 12 | 45 | 2 | 2 | 16 | 5 |
| Septemb. | 38 | 16 | 3 | 19 | 19 | 3 | 13 | 2 | 1 | 8 | 0 |
| October | 40 | 32 | 6 | 40 | 10 | 3 | 15 | 1 | 1 | 6 | 2 |
| Novemb. | 84 | 53 | 13 | 81 | 41 | 21 | 102 | 1 | 1 | 8 | 8 |
| December | 75 | 45 | 14 | 82 | 41 | 17 | 98 | 1 | 1 | 4 | 5 |
| Totals - | 544 | 316 | 116 | 634 | 268 | 98 | 508 | 15 | 14 | 102 | 32" |

91.

From the foregoing table, it appears that, exclusive of the more simple cases of fever, in which purgatives and diluents only were prescribed, there came under Dr. Grattan's observation, in the course of the year, 544 patients, in whom the symptoms were more or less complicated.

" Of this number, 316 had pectoral symptoms, for which 116 were blooded from the arm, the total quantity taken being 634

ounces, or five and a half from each patient. In general the quantity directed to be drawn varied from four to six ounces: the same patient was seldom blooded more than once, and very rarely was it necessary to bleed a third time. In 268 patients the head was affected, and 98 were blooded from the temporal artery, the total number of ounces amounting to 508, giving an average of about five ounces from each patient." 92.

Dr. Grattan here introduces a train of excellent practical observations on the fever, according as it is complicated with affections of different organs. When effusion in the *lungs* took place, the preceding inflammation would not bear the usual measures employed in active inflammation.

"In this state of the lungs, the bronchial cells are filled with mucus or phlegm, poured into them by the secretory and exhalent vessels of the inner membrane of the trachea, throughout its various ramifications: or, this state may, and does sometimes, depend on an engorgement, or tumid condition of the sanguineous capillaries, which from paralysis becoming inactive, and hence unable to propel the blood, are distended so as to compress the bronchial cells, thus preventing the admission of air so necessary to perfect the process of sanguification." 102.

As this condition depends, our author believes, on a relaxed and debilitated state of the vessels, general blood-letting is not calculated to effect a cure, there being no arterial re-action present; though, when the respiration is rendered very laborious, and the function of the lungs is embarrassed, either by accumulated blood or viscid mucus, "venesection must be performed to preserve the patient from suffocation."

"But still it should always be remembered, that the object of blood-letting is only to afford temporary relief, until the vessels, by recovering their usual energy, shall be enabled to maintain the balance of the circulation. If large quantities of blood are taken from a patient in this state, effusion goes on more rapidly, and the fatal termination is of course accelerated. In such cases active purgatives are inadmissible, and for the same reason that blood-letting is objectionable." 103.

In such dangerous cases we must endeavour to relieve the lungs of their load of mucous secretion, by expectorants, given to an extent sufficient to produce not merely nausea, but *retching*.

"The inhalation of the vapour, either of plain water, or of water mixed with vinegar, or ether, sometimes affords great relief, and should be used when the debility of the patient is not such as to prevent its employment." 103.

Dr. Grattan directs that blisters should be applied in succession to the chest; and from the commencement, small doses of calomel and ipecacuanha, at short intervals, and continued with very little interruption, until the symptoms appear to yield. "This they will almost uniformly be found to do, the moment the system is under the influence of mercury." Other auxiliaries are, of course, to be employed, as the practitioner may deem proper; particularly the carbonate of ammonia, digitalis, and even cordials.

Even when acute inflammation of the lungs became complicated with epidemic fever, our author has not found that the large and decisive bleedings, proper in idiopathic pneumonia, were safe. In Dr. Grattan's practice, "the cure was effected more safely, and with equal certainty, by moderate bleedings, assisted by other remedies, than when attempted by copious venesection."

Where the violence of the disease fell principally on the head, the following practice was adopted by our author:

"In all cases where the sensorium is affected, the head should be shaved; if there is an increase of heat, it should be washed with tepid vinegar and water, so as to preserve the head moist, and increase evaporation. When there is delirium, with a flushed countenance, and unusual throbbing of the temples, or pain of the head, although without delirium, the temporal artery should be opened, five or six ounces of blood taken, and a blister applied to the occiput and nape. In the low delirium, unattended with pain of the head, or fulness at the temples, and when the heat of the forehead is not increased, and the face is pale, and the features hollow and contracted, I have been occasionally tempted to try the effects of blood-letting; but I confess I have not found it to answer. In such cases as 4, 20, and 24, of which the two first were not blooded, and in the last of which leeches only were applied, I am not clear that blood-letting is likely to be of use. In many similar instances I have witnessed the recovery of patients under a treatment very different from that of the depleting system. Wine and other stimulants moderately given, camphor, barm, and the acid mixture, gentle aperients, but by no means active or drastic purgatives, successive blisters, and calomel, with a view to affect the system, will often rescue our patient from impending death. However, it will sometimes happen, that when stimulants are required to support the general tone of the vascular system, moderate topical blood-letting may be highly judicious, and even necessary to relieve local congestion. Thus, there are cases in which it will be proper both to bleed from the temples and to give wine, a mode of practice neither as incongruous with itself, nor as inconsistent with a rational theory of the disease as might at first sight appear." 112, 113.

Dr. Grattan found that, where delirium prevailed to such an extent as to preclude sleep, after the head had been shaved, the temporal artery opened once or twice, the nape of the neck blistered, and purgatives administered, that an anodyne of twenty-five or thirty drops of tinct. opii, with a similar quantity of the vinum ipecacuanhæ, repeated every third or fourth hour, until sleep was procured, produced marked beneficial effects, especially towards the decline of the disease.

"After a few hours, tranquil sleep thus obtained, the patient will often, at the next visit, be found free from every alarming symptom, the wild expressions of his countenance will have disappeared, and the convulsive tremors will have become less violent, or have entirely subsided. So far as regards the use of opium in such cases, I have not observed that its employment was followed by any unpleasant effects; I am, however, in the habit of combining it with a diaphoretic, to prevent any injurious consequences which might be supposed likely to proceed from its narcotic properties. In directing opiates to such an extent as thirty drops, repeated every third hour until sleep is procured, it may appear that I venture upon rather large doses; but this will not often prove to be the case, for when the system has been prepared for it by the previous employment of blood-letting and purgatives, the first draught will, in general, be found to produce the desired effect." 118.

In cases of the kind which our author has described, he says "mercury should be freely given. The moment the mouth is rendered sore the symptoms become less violent, and the patient thenceforth gradually recovers." He depends more on the internal administration of the medicine than its external.

"I ascribe its efficacy to its action on the chylopoietic viscera, and more particularly to its power of equalizing the circulation, and removing congestion in the hepatic system, with which the brain unquestionably sympathizes." 119.

The mineral acids, barm, and camphor mixture, though subordinate to more active remedies, are not to be despised. Many cases will occur in which they will be found most valuable auxiliaries.

When pain, fulness, and tension of the abdomen happened to be combined with bad cases of fever, Dr. Grattan knows of no remedy more generally successful in relieving these symptoms than the oleum terebinthinæ, added to the common oil draught.

It is now high time to turn to Dr. Crampton's work. But here, as in the case of Dr. Grattan's Report, we are forced to pass over a great mass of matter, very important to those on the spot, but not equally so to all our readers, scattered as they are, throughout the various climates of this globe. Like KNIGHTS ERRANT of old, too, we have taken a certain vow to pursue a certain object, under every circumstance, and without suffering ourselves to be drawn from our direct course, even were the golden apples of Hippomenes to glance across our path. That object is GENERAL PRACTICAL UTILITY. This is the star that guides us—the magnet that attracts us—the power that impels us. With *this*, for its motto, humble industry is successful; without *this*, the most laboured research is useless.

Speaking of the contagious nature of this epidemic, Dr. Crampton does not pretend to say that *all* those admitted into his wards were cases of contagious fever; on the contrary, he believes that at least one half of them were of a different description.

"Many merely had the symptomatic fever which accompanied inflammatory attacks of the different organs, and which no person conceives to be contagious; in others, fever made its appearance in consequence of the peculiar concurrence of causes to which the poor at this juncture were exposed, independent, I am inclined to think, of any contagious source; yet these latter fevers might be communicated from one individual to another, although they were distinct in their origin; and in the advanced periods of these two kinds of fevers it was quite impossible to distinguish the one from the other." P. 22.

Dr. Crampton having thrown his observations principally into Quarterly Reports, descriptive of the varying types of the epidemic, and the necessarily varying modes of treatment, we cannot follow him into these subdivisions; but keep to more general circumstances. The following passage expresses, and well expresses, what our own experience had long convinced us was the true state of things in all fevers, and in all climates.

"But the destructive changes induced by the fever were not confined to the head. In fact the disease fell with most violence on whatever organ was least able to bear its concentrated attack; according, therefore, as a patient had unsound lungs, a weak stomach, or a diseased liver, the complicated disease had in a great measure the seat of its chief attack predetermined." 44, 45.

Dr. Crampton states here an interesting fact, namely, that "where *a number* of important organs were pressed at the same time, as the brain, the lungs, and the stomach, the danger was less than when the whole force of the disease fell upon *one*, with undivided and concentrated violence. In such cases, provided patients were admitted at early periods, it was satisfactory to see the advantages resulting from an active and energetic practice, and how soon they became convalescent." There was one form of fever which particularly attracted Dr. Crampton's notice; namely, that with which the nurses and the attendants on the sick were attacked.

"It commenced with giddiness or rather staggering, dull headache, which afterwards became intense, loss of appetite, and a white chalky tongue. Many thus circumstanced apprehended they had merely caught cold, a degree of *coryza*, or running at the nose, having in some instances occurred; some felt better the next day, on taking a little medicine, and were averse to lie down for a few days; they felt less inconvenience in the erect posture and in the cool ward than in bed: after this preliminary stage a rigor sometimes took place, at other times it was not observed. The pulse was not always accelerated at this stage, but it was small and indistinct; the skin looked sallow, nor were the alvine secretions much disturbed. A patient thus circumstanced was in imminent danger, severe determination to the head or congestion, to use the language of Armstrong,* having occurred. I do not pretend to say whether this congestion or determination was *venous* or *arterious* in the first instance. That it was not always *venous*, appeared clearly from the inspection of the cerebral organs of the greater number who died of this form of fever, inflammation of the brain and its membranes having often followed the symptoms just mentioned. In other instances, that there was no inflammation was equally evinced by the appearances after death, no change of structure being observed; in a great majority, however, of the fatal cases, where determination to the head existed previous to death, fulness of the arteries of the brain, and marks of inflammation were discernible. After the symptoms, just described, had continued a few days, a violent degree of re-action took place, with a complete development of febrile excitement; and on the third, or at all events on the fourth or fifth day, a patient thus attacked was not unfrequently beyond the reach of medical treatment; delirium, a fixed and glassy eye, loss of senses, loss of speech, and of the power of swallowing, immediately set in. The majority of such patients had petechiæ in different forms; either a mottled or marbled skin, or the measley efflorescence, or the distinct dark petechial ecchymosis." 47.

Another appearance, not mentioned elsewhere, was observed in the late epidemic by Dr. Crampton; viz. "a slight branny desquamation of the cuticle, especially after a long and protracted disease. These patients were very liable to relapses, against which the tepid bath was found the best preservative."

"With patients in the form of fever above described, wine and cordials at the early periods, notwithstanding the excessive prostration of strength, were always given, with the hazard of accelerating the fatal event." 48.

---

"* Armstrong's Practical Illustration of Typhus Fever."

In the onset of such attacks, a full bleeding from the arm, or temporal arteriotomy, afforded a better prospect. The nurses themselves were so well convinced of this, that they always importuned our author for depletion, from ocular demonstration of its utility. Shaving the head, and the cold affusion, after bleeding, rendered the future progress of the fever more tractable. It was next to impossible, to bring the fever, in these cases, to a sudden termination, by any means that were tried.

"Medical assistance went chiefly to prevent destructive changes of structure in important organs. With those, however, who immediately submitted to active treatment, the disease became mild in a few days, and there are a few instances where the symptoms subsided at once."

Purgatives and tepid affusions were used; the former constantly, the latter when the weather was cold. In a fever ward of children, Dr. Crampton employed the warm bath in all stages of the fever, and during convalescence; with the effect of shortening the one and securing the other. We have witnessed the powerful effects of the warm bath in diseases of children so often, that we scarcely know a complaint, in this class of patients, which is not benefited by it. In children, the skin seems torpid, compared with the activity of the internal organs, in health;—in disease, therefore, it is a great object to increase the excreting function of this great surface, particularly by the warm bath. We can very readily credit the following assertion.

"When venesection was used during the first few days of this fever, the pulse, from having been small and feeble, expanded, and became more full and sensible to the touch. If a compromising line of practice was adopted, and if patients were blooded, not to the extent that afforded relief, or else, if it was at periods too advanced for the strength or vital powers to bear, a want of success was the result, and discredit was thus brought on the practice." 50.

When relapses occurred, with severe symptoms, the lancet, in general, was found to be the best remedy, the blood in such cases being, for the most part, buffy; whereas, on the first onset of the fever, this appearance was wanting. The convalescence from this fever, even after repeated relapses and great prostration of the animal powers, was often surprisingly quick, unless where there was some constitutional disease in complication with fever.

"The disease mostly proved fatal to such as had been given wine or distilled spirits before they were carried to the hospital; a kind of *delirium tremens* accompanied the fever, and succeeded it if they happened to surmount its attack, and a nervous and anomalous train of symptoms became established for a long time afterwards." 51.

Dr. Crampton judiciously observes that, although mild cases, in general, recover under almost any mode of practice; yet many, whose diseases were rendered moderate by the favourable circumstances of cool wards, clean and comfortable beds, diluents, and early medical assistance, very soon exhibited a different character, if allowed to remain in heated and close rooms, "where infection is concentrated, and where proper treatment is neglected." Indeed, no case of fever can be said to be so mild as not to require constant medical superintendance.

"Even under the most auspicious circumstances, affections of the brain and other important organs are apt to supervene at all periods of fever; which, if neglected, soon put the patient out of the reach of medical assistance."

Shaving and washing the head afforded great advantages, not only in preventing determinations to the brain, but in facilitating the adoption of other measures at subsequent periods, such as the application of leeches, blisters, and refrigerating lotions.

On the subject of *post mortem* appearances, we shall introduce the following passage from our experienced and able author.

"The appearances which I observed in the brain and its membranes, were fulness and distention of the vessels, the arteries as well as the veins, the former often appearing as if subjected to an anatomical injection; extravasation of blood was seen in a few instances, sometimes under the arachnoid, and again at the base of the brain; effusion into the ventricles and under the arachnoid was frequently witnessed, as well as into the spinal canal; a thickening and opacity of the arachnoid and of the pia mater was not an unusual occurrence, and lymph was often thrown out, sometimes a wheyish fluid, and again a gelatinous substance. These membranes occasionally manifested more unequivocal signs of previous inflammation, exhibiting their surfaces superficially ulcerated and covered with a purulent discharge. Some other varieties in the appearances found in the brain were observed, but they were mostly of the same character." 54.

Our readers are aware, that Drs. Percival, Cheyne, Mills, Bateman, and Duncan, (Jun.) corroborate the above statement; but the testimonies of Dr. Macartney and Mr. Kirby are adduced by Drs. Barker and Stoker to maintain a somewhat different opinion.* We shall not enter into any dis-

* See our Fever Article, p. 547, Vol. i, Quarterly Series, April 1819.

cussion respecting these little discrepancies among physicians, who are otherwise so generally agreed upon all the leading points both of pathology and practice.

Where the thoracic organs were most pressed during fever, the fatal cases invariably exhibited changes of structure, the unequivocal products of inflammation, as recent adhesions of the pleura, exsudation of wheyish or sero-purulent fluids, effusions of lymph and formation of false membranes, besides analogous changes of structure in the mucous, bronchial, and other textures. Tubercles were often found in the lungs, in every stage of development; nor did the heart and its membranes escape.

"In a considerable number of those examined, the liver was found tuberculated and diseased in a variety of forms; but this is not surprising, when we consider the number of intemperate drunkards, with worn out viscera, who occupy that great depôt of poverty, the House of Industry; and who, when afflicted with febrile symptoms, are from thence transferred to the fever wards. The diseased state of these organs could not, with propriety, be regarded always as the only cause of fever, although it was certainly instrumental in hastening the death of the patient. In many instances, however, the serous membranes covering the liver were found with fresh coatings of coagulable lymph; serous effusions and recent adhesions were also observed; and these appearances could be connected with severe local pains, and other symptoms of inflammation, which must have existed shortly antecedent to death." 57.

In the gastric form of fever, the *mucous* coat of the stomach, and sometimes of a portion of the intestines, was found slightly inflamed. When the symptoms denoting this state were neglected, the inflammation spread to the peritoneal coverings.

In the dysenteric forms of fever, inflammation was present at some stage of the disease, and erosion and ulceration were the result. On the subject of a tuberculated or warty state of the villous coats of the intestines, especially in children, we have given an account, in the second volume of our last Series, p. 267.

"The anatomical preparations made at the House of Industry, form an incontestible chain of evidence which ought to convince the most sceptical, that in the severe forms of the fatal cases of fever, there is something more than *venous congestion* to be attended to." 58.

Most undoubtedly there is; and those who have described these states of venous congestion as sometimes happening, are well aware, that the states of arterial excitement are much more frequently met with.

"It is very possible in the onset of fever, that congestion or distention of the vessels of the brain, in addition to other derangements of the cerebral system may be present, but that it is by no means limited to the venous system; this congestive state, however, soon passes into the inflammatory in the severe cases, unless measures are taken to prevent it. Far be it from me to say, that inflammation is the cause of fever, or that it is necessarily present in every instance, the contrary I believe is the fact in the great majority of the mild cases; but the appearances mentioned, and the important changes of structure induced, warrant me in asserting, that in the greater number of severe cases legitimate inflammatory symptoms sooner or later become evolved, and that it is by looking to these that the mortality in fever on a large scale is chiefly to be prevented." 59.

In this sentiment we entirely agree, and with this extract we shall conclude.

Dr. Grattan and Dr. Crampton deserve well of their brethren, and of their country at large, for their zealous exertions in the cause of medical science and of humanity. May the constellation of talent which rises over the Sister Island, diffuse its beams of light, and knowledge, and happiness over every habitable portion of the Globe.

Videmus literas et ingenuas artes non solum beatæ vitæ oblectationem esse, sed etiam levamentum miseriarum." CICERO.

